# Atlas of Brain Glucose Metabolism Using Deuterium Metabolic Imaging at 3 T

**DOI:** 10.1002/mrm.70451

**Published:** 2026-05-30

**Authors:** Kamilla K. Trosborg, Nichlas V. Christensen, Malene Aastrup, Nikolaj Bøgh, Janne K. Mortensen, Hanne Gottrup, Per Borghammer, Mattias H. Kristensen, Esben S. S. Hansen, Sofie H. Christensen, Jack J. Miller, Rolf F. Schulte, Michael Vaeggemose, Lotte B. Bertelsen, Christoffer Laustsen

**Affiliations:** ^1^ The MR Research Centre, Department of Clinical Medicine Aarhus University Aarhus Denmark; ^2^ Department of Clinical Medicine Aarhus University Aarhus Denmark; ^3^ Department of Neurology Aarhus University Hospital Aarhus Denmark; ^4^ Department of Nuclear Medicine and PET Centre Aarhus University Hospital Aarhus Denmark; ^5^ Department of Mathematics Aarhus University Aarhus Denmark; ^6^ GE HealthCare Munich Germany; ^7^ GE HealthCare Brøndby Denmark

**Keywords:** Alzheimer's disease, atlas, brain, deuterium, glucose metabolism, magnetic resonance imaging

## Abstract

**Purpose:**

Deuterium metabolic imaging (DMI) offers noninvasive magnetic resonance imaging (MRI)–based assessment of metabolism in vivo, making it a relevant paraclinical tool for diseases with neurological metabolic alterations. This study aimed to establish a normative reference atlas of brain glucose metabolism accounting for age and sex.

**Methods:**

DMI were obtained for 30 healthy adults (aged 51–84 years, 15 female) with a 3 T MRI scanner after ingestion of deuterated [6,6'‐^2^H_2_]glucose. The images were parcellated to determine the regional distribution of deuterated water, glucose, lactate, and glutamate plus glutamine (Glx). Linear models were applied to investigate the effects of age, sex, and other exploratory adjustments. As a proof‐of‐concept example of atlas application, the normative atlas was compared with patients with Alzheimer's disease and healthy subjects from a previous study.

**Results:**

Regional differences were significant for all metabolites (*p* < 10^−15^), with the highest values in the occipital lobes, except for lactate, whose regional distribution pattern was less consistent. While lactate production showed no overall age‐dependency, global Glx production decreased 13% ± 4% per decade. Lactate production tended to be higher in males than females (*p* = 0.042), but this was not significant after regional adjustment (*p* = 0.084). Discriminating between health and Alzheimer's disease required additional adjustments for weight, blood glucose, and timing.

**Conclusions:**

While regional and age effects explained a substantial part of the variability in Glx, reliable intersubject comparisons required additional adjustments. The normative atlas presented here provides a reference for future DMI studies of brain metabolism.

## 1 Introduction

Glucose is the principal energy source for the human brain [1], and impairment of brain glucose metabolism plays an important role in several diseases including type 2 diabetes [2], cancers [3], and neurodegenerative disorders such as Parkinson's disease [4] and Alzheimer's disease (AD) [5]. As these diseases are most frequently developed late in life, their prevalence is increasing along with the growth of the elderly population [6, 7]. This triggers a rising demand for paraclinical tools to investigate, diagnose, and monitor these diseases [8].

As of now, ^18^F‐fluodeoxyglucose positron emission tomography (FDG PET) is the most widely used method for in vivo studies of human brain glucose metabolism [9], and it is part of the current European recommendations for the diagnostic investigation of various cancers [10] and neurodegenerative disorders including AD [11].

Deuterium metabolic imaging (DMI) is a novel nonionizing magnetic resonance imaging (MRI) technique that may generate insight beyond that of FDG PET, since measurements are not limited to glucose uptake but also encompass downstream metabolites [12, 13]. The glucose analogue [6,6'‐^2^H_2_]glucose used for DMI is metabolized intracellularly, and by spectrally resolving the DMI signal, it is possible to distinguish the signals originating from deuterated water (HDO), glucose, and lactate, as well as the compound signal from glutamate and glutamine (Glx) [14]. This ability to separate aerobic glycolysis in the cytosol (lactate) from oxidative phosphorylation in the mitochondria (Glx) could potentially improve diagnostic and prognostic specificity. DMI can be implemented at already existing 3 T clinical systems, thereby enabling the acquisition of both metabolic images and conventional structural images in a single scan session [15]. Furthermore, the deuterated glucose is a stable isotope produced as an off‐the‐shelf product that can be easily transported, stored for months and ingested orally, thus simplifying the clinical procedure [12, 16].

Accordingly, DMI holds the potential to become a safe, simple, readily available, and affordable paraclinical tool for investigation, diagnosis, and monitoring of diseases with altered brain glucose metabolism. Like other MRI and metabolic imaging methods, realizing the potential of DMI requires a normative atlas for researchers and clinicians to refer to when evaluating patient scans [17, 18, 19]. Such an atlas has never been established in any previous DMI studies of brain glucose metabolism at a clinically relevant field strength; the maximal number of healthy humans included in previous studies count only nine [20, 21].

Since FDG PET studies show that brain glucose uptake and metabolism decrease with age [22] and depend on biological sex [23, 24] and brain region [25], we hypothesized that brain glucose metabolism assessed with DMI exhibits similar age, sex, and regional differences which should be taken into account when constructing a reference atlas.

Therefore, the aim of this study was to establish a normative reference atlas of human brain glucose metabolism in an elderly population based on DMI while taking the effects of age, sex, and region into account and exploring secondary parameters to enhance atlas performance.

## 2 Methods

### 2.1 Study Design and Participants

This prospective clinical study was approved by the Ethics Committee of Central Denmark (1‐10‐72‐80‐24). A total of 30 healthy volunteers (HC), comprising 15 of each sex, were recruited through public advertisement.

Inclusion criteria were 50–85 years old and able to provide written informed consent.

Exclusion criteria were MRI contraindications (i.e., pacemaker, neurostimulator or cochlear implant; metal foreign bodies such as fragments and irremovable piercings; unsafe medical implants; claustrophobia; maximum body circumference including arms > 160 cm) along with metabolic diseases such as diabetes or hypothyroidism, and neurological conditions such as tumors, epilepsy, previous stroke, chronic small vessel disease (defined by Fazekas score ≥ 2 if clinical MRI was available), or neurodegenerative or neuroinflammatory diseases. Additionally, women had to be post‐menopausal or confirmed non‐pregnant by an onsite test.

As a proof‐of‐concept example of atlas application, we retrospectively analyzed data from 10 patients with AD and five HCs included in a previously published paper [15]. These volunteers were included according to the same criteria as described above with the exception that patients with AD were required to be newly diagnosed with AD according to the National Institute on Aging and Alzheimer's Association 2011 research criteria [26] and have a mini‐mental state examination score of ≥ 18 [27]. Patients with AD were recruited from the Department of Neurology, Aarhus University Hospital, Denmark.

### 2.2 Imaging

**FIGURE 1:**
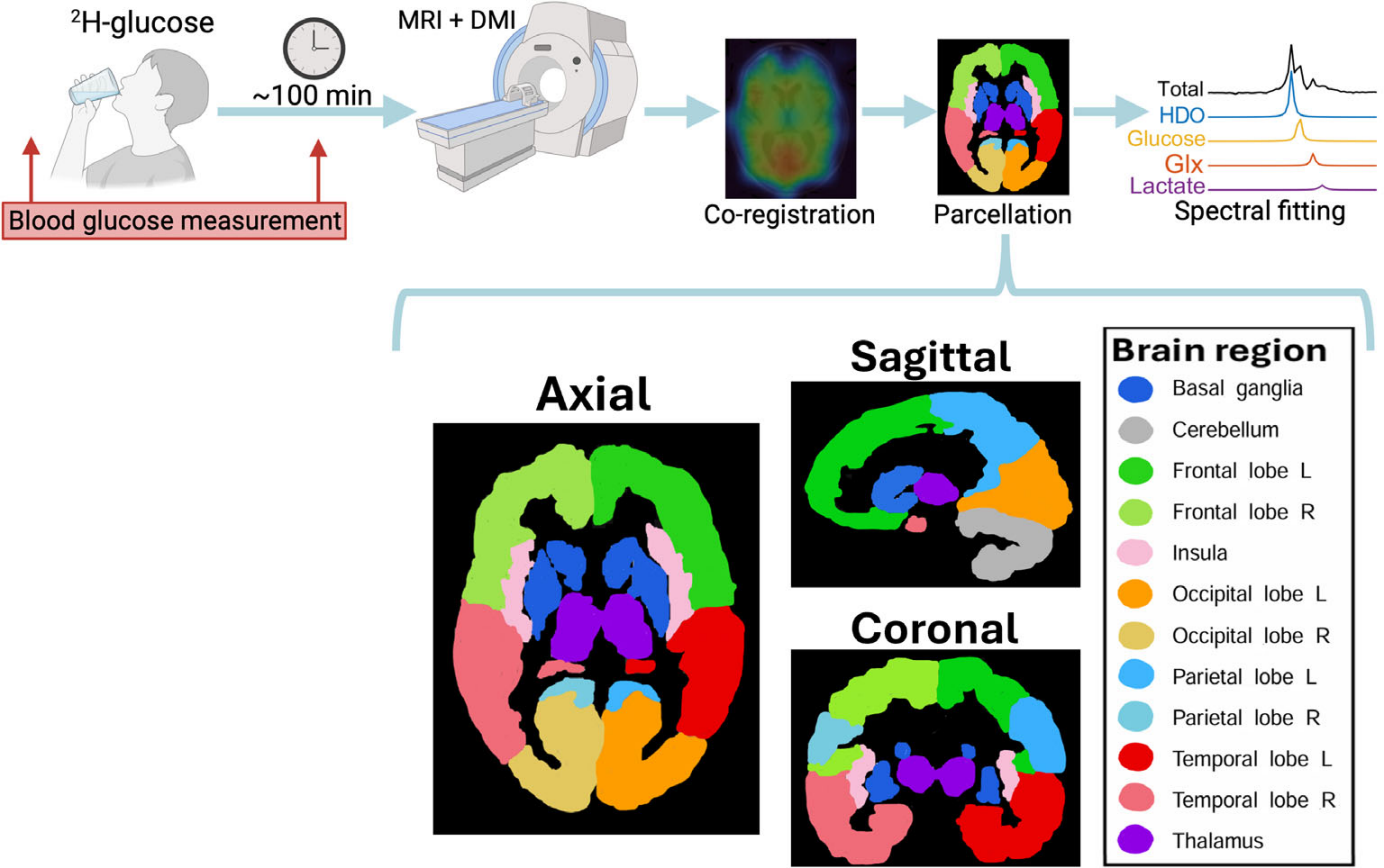
Study workflow. The deuterium metabolic imaging (DMI) scan was performed about 100 min following glucose ingestion with blood glucose being measured at baseline and before the scan. The images from DMI and anatomical MRI were co‐registered to extract the DMI spectra from the chosen brain regions, before these spectra were fitted to yield the signal intensities of individual metabolites: Deuterated water (HDO), glucose, glutamate plus glutamine (Glx), and lactate. The parcellation of a healthy brain is displayed in axial, sagittal, and coronal planes. While the brain lobes (frontal, parietal, temporal, and occipital) were divided into left (L) and right (R), the cerebellum and smaller brain regions (insula, thalamus, basal ganglia) were not. Part of the figure was created in BioRender. Laustsen, C. (2026) https://BioRender.com/kvpfwep.

The study workflow is illustrated in Figure 1. All participants fasted for at least 4 h, and after a measurement of baseline blood glucose with a point‐of‐care device (Contour, Bayer Consumer Care) they ingested 75 g of [6,6′‐^2^H_2_]glucose (Cambridge Isotope Laboratories Inc.) dissolved in 200 mL tap water. They were then asked to keep activity at a minimum and refrain from talking and reading for about 50 min before the second measurement of blood glucose and subsequent entry into the MRI scanner. We used a 3 T clinical MRI scanner (MR750, GE HealthCare) equipped with a dual‐tuned proton‐deuterium birdcage head coil (PulseTeq) and aimed at starting the DMI scan 90–105 min after glucose ingestion.

Before DMI, anatomical proton imaging was performed using the BRAVO sequence. This is a T_1_‐weighted 3D inversion‐recovery prepared fast spoiled gradient echo sequence (resolution = 2 × 2 × 2 mm^3^, flip angle = 12°, repetition time = 5.5 msec, echo time = 1.7 msec, inversion time = 450 msec, number of acquisitions = 2, scan time = 4 min and 27 s) [28].

The DMI data were acquired with 3D magnetic resonance spectroscopic imaging (MRSI) using a hard pulse (flip angle = 70°, repetition time = 155.8 msec) and a density‐weighted phase encoding (effective matrix size = 10 × 10 × 10, nominal matrix size = 16 × 16 × 16, field of view = 24 × 24 × 24 cm^3^, echo time = 0.7 msec, number of acquisitions = 4, spectral points = 700, bandwidth = 5 kHz), resulting in a total scan time of 17 min and 26 s (Table S1).

### 2.3 Image Processing

The DMI spectral images were reconstructed in MATLAB (version R2024b; MathWorks [29]) using the MNS Research Pack (GE HealthCare). Voxels with an SNR < 5 of the maximal peak (HDO) were excluded from further analysis before denoising performed using tensor‐based Marchenko‐Pastur principal component analysis (tMPPCA) [30] with window size [3] followed by averaging the four signal acquisitions. Partial volume correction (Richardson‐Lucy iterative deconvolution) was applied to improve sharpness followed by bias field correction using multiplicative intrinsic component optimization (MICO) [31]. Representative spectra of raw and processed data are presented in Figure S1.

DMI MRSI were subsequently co‐registered to the T_1_‐weigthed images using SPM12 [32] and normalized using affine and nonlinear registration to the MNI152 standard space [33], before parcellation with an automated labeling atlas [34]. The labeling atlas contains 170 brain regions. These were summed into bigger parcels to construct the brain lobes (frontal, parietal, temporal, and occipital), which were divided into left and right, while the cerebellum and smaller brain regions (insula, thalamus, basal ganglia) were not divided into a left and right part. Accordingly, 12 different brain regions were used in the analysis (Figure 1). These large composite regions were used to minimize the risk of partial volume effects that arise because the inherently low SNR of DMI at 3 T constrains the spatial resolution. Additionally, part of the analysis was performed on the “whole brain” which refers to the sum of all areas of the brain, including but not limited to the 12 regions described earlier. Following atlas construction, the fractional coverage per DMI voxel of each atlas region within the corresponding T_1_‐weighted block was computed and used as linear weights to aggregate signals into the respective region spectra before fitting.

DMI region spectra were time domain fitted using a MATLAB adaptation of the Advanced Method for Accurate, Robust, and Efficient Spectral fitting (AMARES) algorithm as described elsewhere [35, 36] to determine signal components of the individual metabolites: HDO, glucose, Glx, lactate, and lipid. Note that lipid was fit in conjunction with the other metabolites to account for overlap with lactate, but was not used for further analysis. All regions with HDO linewidth > 30 Hz were excluded from further analysis. Representative region spectra fits are presented in Figure S2.

For probing the conversion of glucose into products of oxidative phosphorylation (Glx) and aerobic glycolysis (lactate), the signals of each of these metabolic products were normalized to the combined glucose and HDO peaks. The combined signal was chosen (as opposed to, e.g., only the glucose peak) to circumvent potential separation errors caused by the fact that glucose and HDO signals are spectrally overlapping, thus making the combined peaks a more robust normalization reference.

### 2.4 Statistical Analysis

All statistical analyses were performed in R (version 4.4.2; R Core Team [37]), except for a post hoc analysis of statistical power calculated with G*Power (version 3.1.9.7; Faul et al. [38]). A *p* value of < 0.05 was considered statistically significant. The assumption of normality was confirmed by Q–Q plots and by scatterplots of residuals against predicted values from linear regression models.

The effect of age and sex on Glx and lactate normalized to the combined glucose and HDO signals was investigated with linear regression.

To assess regional differences, the mean metabolic values of each brain region were converted into z‐scores, defined as the number of standard deviations (SDs) from the mean of all brain regions within a single subject. This was done for all four metabolites.

To combine regional differences as well as age and sex in a common model, linear mixed‐effects regression was applied (using the lme4 package in R [39]) with participant ID included as a random factor [40]. Building the models was an iterative process, where models of increasing complexity with region, age, sex, and their interaction terms were compared. The final parsimonious models were chosen based on ANOVA comparisons, Akaike information criterion (AIC), and explained variance (*R* ^2^) (Table S2). In an exploratory analysis, several additional variables were included in one comprehensive model. Here, the explanatory variables with a potential association to the DMI outcomes were identified based on biological plausibility and a correlation matrix (Figure S3).

Model performance was evaluated using parametric bootstrapping with 1000 simulations to create predictions and prediction intervals. For internal validation, the model data itself (30 HCs) was used to generate predicted values which were plotted against the observed values, and correlations were computed using the Pearson approach, while the prediction accuracy was assessed with mean absolute error (using the Metrics package in R [41]) (Figure S4). For external validation, we used test data from a previous study including 10 patients with AD and five HCs. First, the AD group and HC group from the test data were directly compared with the 30 HCs from the normative atlas using type III ANOVA while adjusting for the factors included in the models. Second, the predictions generated from parametric bootstrapping of the test data were used to illustrate external model performance by calculating the number of SDs that the predictions differed from the observed values.

### 2.5 3D Brain Illustrations

3D rendering of brain atlases was done in Python 3.11 using Pyvista [42]. The cortical part of the atlas was rendered using Nilearn's *fsaverage5* template, whereas the cerebellum was rendered using a hierarchical atlas from Nettekoven et al. [43].

## 3 Results

### 3.1 Participants

Participant characteristics are summarized in Table 1. A total of 30 healthy adults (mean age 64.3 ± 11 years, range 51–84 years) were included. During the recruitment process, seven volunteers were excluded based on the exclusion criteria (one male and three females due to metabolic diseases, one female due to neurological disease, two females due to MRI contraindications) and recruitment was continued until the planned number of 30 eligible volunteers was reached. The 15 males and 15 females were well matched on age, BMI, baseline blood glucose, and waiting time from glucose ingestion to DMI scan. Males were significantly taller (*p* < 0.001) and heavier (p < 0.001) than females and had a significantly greater post‐ingestion increase in blood glucose compared with females (*p* = 0.028).

**TABLE 1:** Participant characteristics.

Characteristic	Total (n=30)	Male (n=15)	Female (n=15)	*p*
Age (years)	64 ± 11 (51–84)	65 ± 11 (51–84)	64 ± 12 (51–84)	0.79
Height (cm)	173 ± 10.1 (150–193)	181 ± 6.16 (168–193)	165 ± 6.22 (150–176)	< 0.001
Weight (kg)	73 ± 11 (46–95)	80 ± 7.8 (70–95)	66 ± 9.9 (46–79)	< 0.001
BMI (kg/m^2^)	24 ± 2.5 (18–29)	25 ± 2.4 (20–29)	24 ± 2.7 (18–28)	0.55
Blood glucose at baseline (mmol/L)	5.3 ± 0.44 (4.6–6.4)	5.4 ± 0.44 (4.6–6.3)	5.2 ± 0.46 (4.6–6.4)	0.47
Blood glucose increase (mmol/L)	4.2 ± 1.6 (0.50–8.2)	4.8 ± 1.7 (1.4–8.2)	3.6 ± 1.3 (0.50–5.5)	0.028
Waiting time from glucose ingestion to DMI scan (min)	94 ± 7.8 (88–128)	95 ± 9.7 (89–128)	93 ± 5.6 (88–106)	0.67

*Note:* Data are means ± SDs, with ranges in parentheses. *p* = *p*‐value for *t*‐test comparing males and females.

Abbreviation: BMI, body mass index.

### 3.2 Age and Sex Differences in Glx and Lactate Production

**FIGURE 2:**
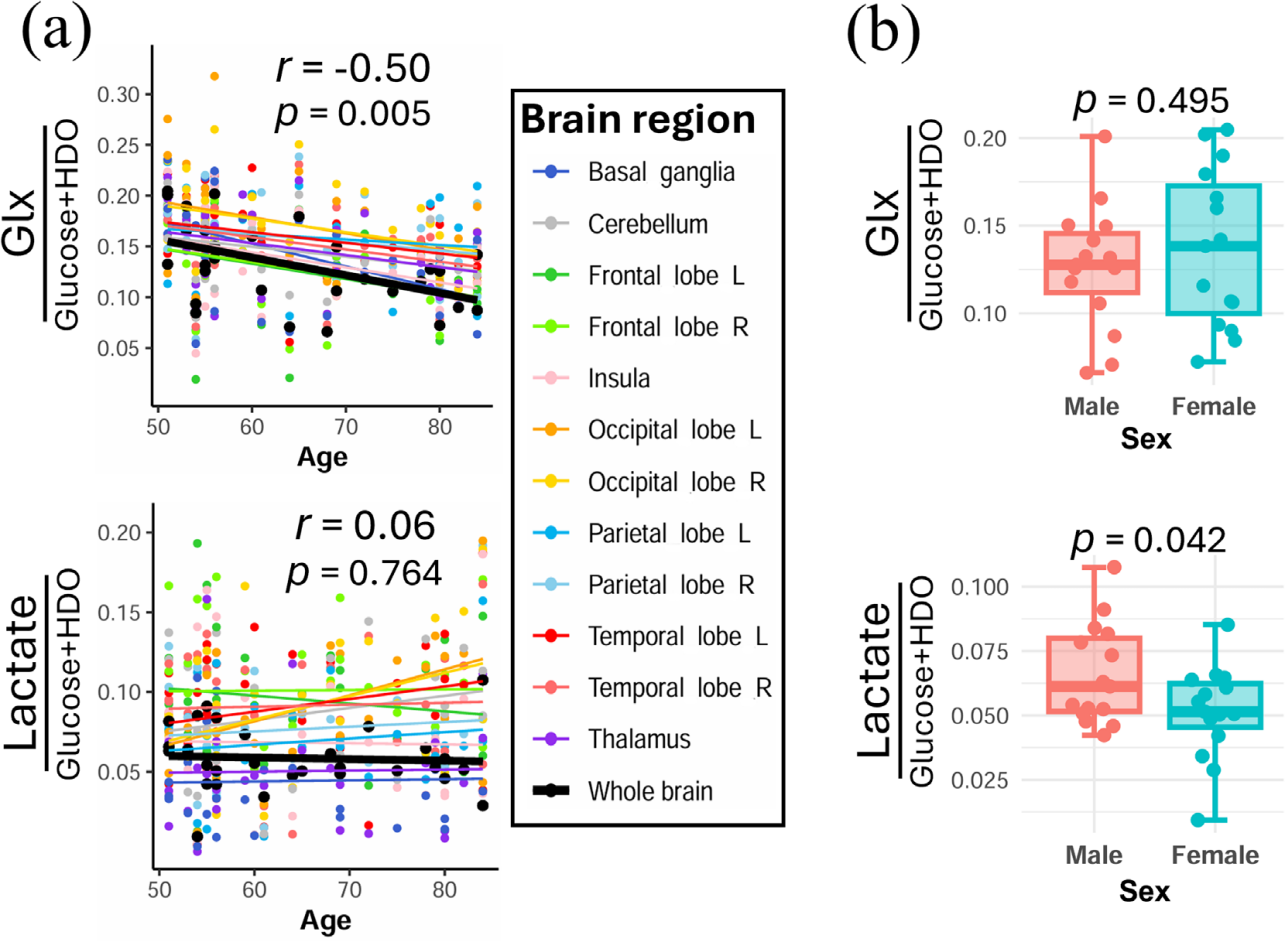
Age and sex differences in Glx and lactate production. (a) Glutamate plus glutamine (Glx) and lactate normalized to glucose and water (HDO) are shown as a function of age. Each color represents a different brain region, while the whole brain is marked with black. (b) Boxplots showing the normalized Glx and lactate for each sex. In both (a) and (b), the *r* and *p* values above the plots are from the whole brain analysis, and all numbers are unadjusted. L, Left; R, Right.

In simple linear models, age was significantly associated with Glx production Glx/(Glucose+HDO) with a global decline of 13% ± 4% per decade relative to the mean (*p* = 0.005, *r* = −0.50, statistical power = 0.86) (Figure 2a). No age association was observed for lactate production Lactate/(Glucose+HDO), except for a significant increase confined to the occipital lobes (left lobe: *p* = 0.006, right lobe: *p* = 0.033).

While Glx production did not differ between the sexes (*p* = 0.495), males had significantly higher global lactate production compared with females (*p* = 0.042) (Figure 2b).

Adjusting for sex in the age analyses and for age in the sex analyses did not change the significance status or magnitude of reported measures. However, additional adjustment for brain region abolished the significance of the sex term for lactate (*p* = 0.084).

### 3.3 Regional Differences in HDO, Glucose, Glx, and Lactate

**FIGURE 3:**
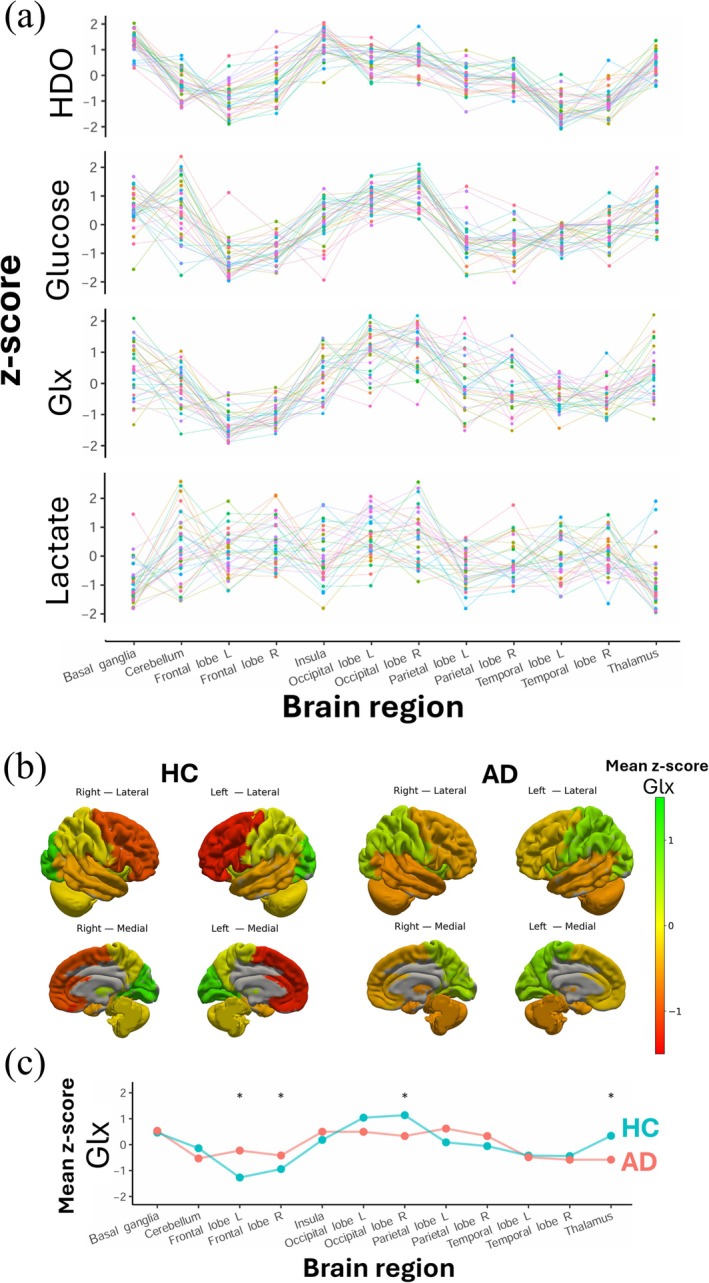
Topography of z‐scores for glucose metabolites. (a) The regional z‐scores for deuterated water (HDO), glucose, glutamate plus glutamine (Glx), and lactate, are plotted against the brain region with each color representing a different healthy subject (*n* = 30). (b) Mapped on template brains is the mean Glx z‐scores of the 30 healthy controls (HC) constituting the healthy atlas and of the 10 patients with Alzheimer's disease (AD) from the test data. Areas not included in the atlas are gray. (c) Plots of the same values as in (b). * brain regions with a significant (*p* < 0.05) difference between HC and AD. L, Left; R, Right.

The regional z‐scores for each metabolite are displayed in Figure 3a. As z‐scores reflect relative regional distribution patterns rather than absolute metabolic levels, they were used to compare topographic profiles across subjects and groups. One‐way ANOVAs revealed that the regional differences were significant for all four metabolites (*p* < 10^−15^). While lactate showed a low degree of consistency, z‐scores for HDO, glucose, and Glx were relatively consistent across subjects with low values in the frontal lobes and high values in the occipital lobes.

To compare the normative atlas with other subjects, the mean z‐score of all 30 HCs was used, and similar mean z‐scores were calculated for the retrospective AD data. The resulting mean Glx z‐scores are mapped on template brains in Figure 3b and plotted in Figure 3c. This comparison shows that the distribution of Glx is different for the two groups with ADs presenting a more homogeneous distribution across brain regions, thus having relatively more Glx in the frontal lobes and relatively less in the occipital lobes and thalamus.

### 3.4 Models and Predictions for Glx and Lactate Production

The process of building linear mixed‐effects models with brain region, age, and sex resulted in a final parsimonious model for Glx production Glx/(Glucose+HDO) including region and age, while the model for lactate production Lactate/(Glucose+HDO) included region and sex, both models without interaction terms (Table S2).

The performance of the parsimonious models was limited. Internal validation produced mean absolute errors of about 0.03, corresponding to about 20% error on Glx and 35% on lactate. The correlations between predicted and observed values were weak for both Glx (mean *r* = 0.33) and lactate (mean *r* = 0.24) (Figure S4). In external validation, both models led to a significant difference in metabolic ratios between the 30 HCs from the model data and the five HCs from the test data, and provided no discrimination between HC and AD.

In the exploratory analysis, the following additional variables were identified for inclusion in the comprehensive models: weight, blood glucose increase, and time from glucose ingestion to DMI scan (Figure S3).

**FIGURE 4:**
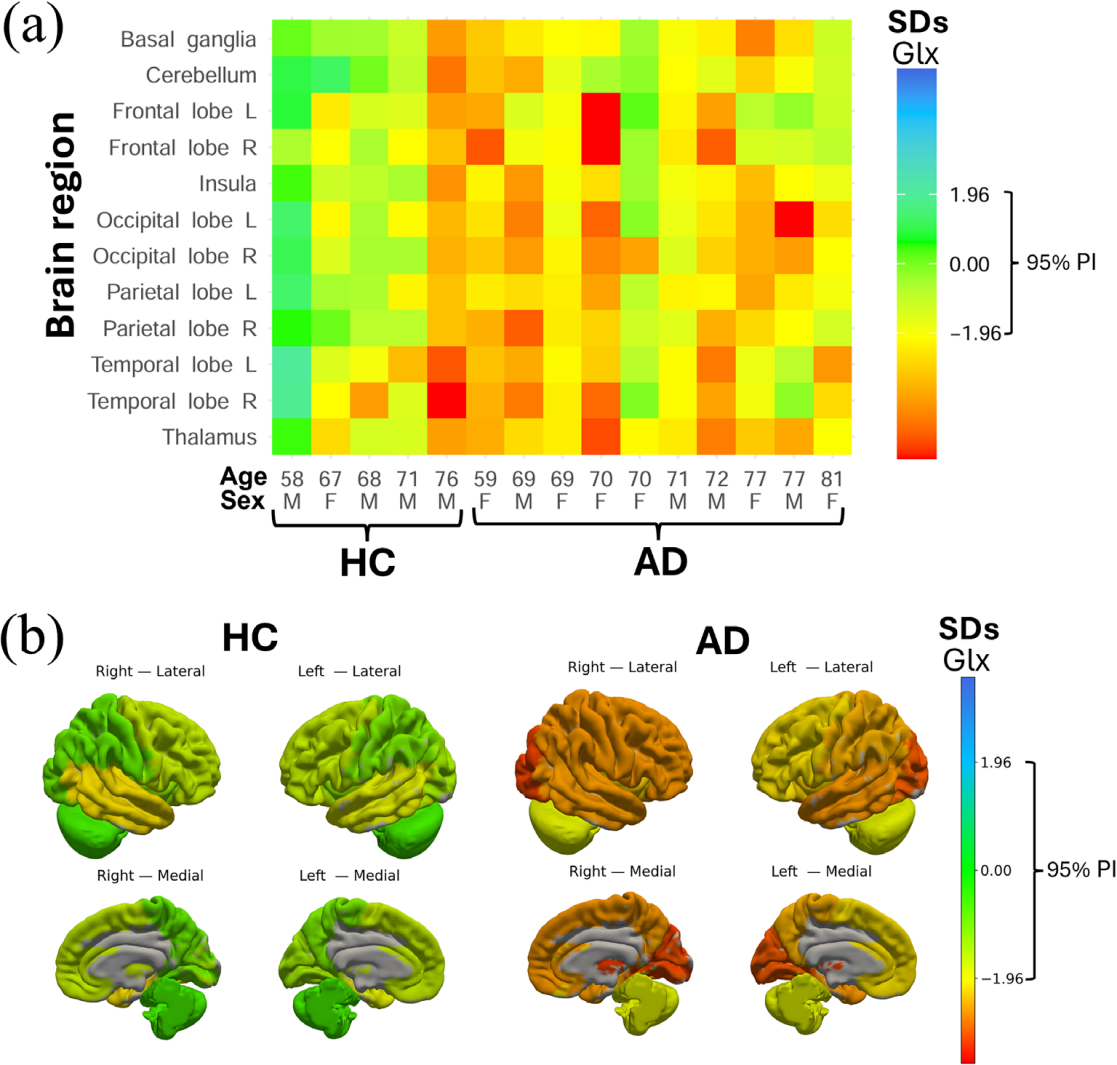
Prediction deviations for Glx production. The color represents the number of standard deviations (SDs) that the observations from the test data differ from the predictions in the model for glutamine plus glutamate (Glx) normalized to glucose and deuterated water. These prediction deviations are shown in (a) for each subject on a heatmap, and in (b) as the mean of the five healthy controls (HC) and the 10 patients with Alzheimer's disease (AD) on template brains. The 95% prediction interval (PI) is marked on the color scales. In all examined brain regions, the HC mean is within or close to the 95% PI, while the AD mean is below except for the cerebellum. Areas not included in the atlas are gray. L, Left; R, Right; M, Male; F, Female.

The comprehensive models demonstrated major performance improvement with respect to Glx (mean *r* = 0.50) and minor improvement for lactate (mean *r* = 0.30) (Figure S4). Furthermore, the comprehensive Glx model was able to discriminate HCs from ADs in the test data (*p* < 10^−7^), since nearly all the observed mean values for the test HCs fell within the 95% prediction interval of the model, while the observed mean values for the ADs fell below the prediction interval in all brain regions except the cerebellum (Figure 4).

Because the model‐building process for lactate proposed an age–region interaction term as a possible predictor (Table S2), this term was included in a post hoc analysis, but did not markedly enhance the performance of the parsimonious nor the comprehensive model (data not shown).

## 4 Discussion

The main outcome of this study was the establishment of a normative reference atlas of brain glucose metabolism using DMI. The metabolism was found to be highly dependent on brain region and age, whereas the evidence of a sex effect was weak. An exploratory analysis revealed that additional adjustment for weight, blood glucose increase, and time from glucose ingestion to DMI scan can enhance the performance of the normative atlas, enabling it to discriminate healthy subjects from patients with AD in a retrospective dataset.

The regional distribution of metabolites identified in this DMI study is comparable with previous FDG PET and carbon‐13 (^13^C) MRI studies. First, FDG PET studies show high levels of glucose uptake and metabolism in the occipital lobe [44], and our study likewise identified this lobe as the brain area with the highest levels of Glx and glucose. Second, in ^13^C MRI, the bicarbonate measured after injection of hyperpolarized [1‐^13^C]pyruvate is a marker of oxidative phosphorylation and thus biologically corresponds to Glx in DMI [45]. Using this ^13^C MRI method, Lee et al. observed the highest bicarbonate levels among the elderly in parts of the occipital lobe, namely the cuneus and lingual gyrus [46].

Among the brain regions inspected in this DMI study, the frontal lobes exhibited the lowest values of Glx and glucose, which contrast FDG PET studies reporting high levels in frontal lobes [44, 47]. This discrepancy might in part be explained by the fact that the FDG PET studies were performed on a younger cohort than the current DMI study because the frontal lobe has been proven to be particularly prone to age‐related changes in glucose metabolism [22, 44, 48]. A strong age dependency of the frontal lobe is furthermore in line with the results of our age analysis, where the frontal lobes showed the steepest decline in Glx production with age. However, even in the old subgroup (aged 65 to 85 years) of their stratified analysis, Andersen et al. find relatively high values in the frontal lobes [44], implying that age differences do not provide a full explanation for the discrepancy. Other explanations might be related to differences in experimental setup. For example, the fact that the frontal lobes are associated with executive functions such as planning, problem solving, and working memory [49], means that the cognitive tasks performed by the participants in the time preceding the scan could have influenced the results.

Alternatively, the low levels measured in the frontal lobes could be related to coil sensitivity and inhomogeneities of the magnetic field, because uniform shimming of the frontal lobe is notoriously difficult due to its close relation to the sinuses and skull [50]. However, since the gyromagnetic ratio of deuterium is 6.5 times lower than that of the proton, deuterium is less prone to the inhomogeneities [12]. Furthermore, even if the hardware‐related signal loss might explain the low z‐scores in the frontal lobes, the signal loss is expected to be of the same relative magnitude for all metabolites investigated, thereby leaving metabolic ratios unaffected. Thus, it does not explain the fact that low values in the frontal lobes and high values in the occipital lobes were observed not only for z‐scores but also for the ratio Glx/(Glucose+HDO).

On the one hand, the use of ratios reduces hardware‐related errors. On the other hand, this normalization approach increases the complexity of variable interpretation by making the variables depend on the denominator as well as the numerator. For instance, the apparent higher Lac/(Glucose+HDO) in males compared with females may be caused by lower glucose rather than higher lactate. This in turn could be the result of differences in the physiological response to glucose ingestion, where males more often than females have a monophasic glucose curve (as opposed to multiphasic) [51], which is associated with insulin resistance [52, 53]. Thus, the fact that males in our study showed a higher blood glucose increase ˜50 min post‐ingestion (Table 1) might be an expression of this monophasic pattern, leading to a reduced brain glucose uptake at the time of the scan. If this is the case, why did we not observe a similar sex difference for Glx/(Glucose+HDO)? An explanation could be that the oxidative phosphorylation and thus Glx production were reduced in parallel with the reduced glucose uptake. In this way, the applied ratios reflect the ability of the brain tissue to convert glucose into Glx or lactate relative to how much glucose is available in the tissue, which is why we refer to the ratio as Glx or lactate “production” in this paper. Since blood glucose increase was not correlated with age (Figure S3), it is not likely to have influenced the interpretation of the age‐related decrease in Glx/(Glucose+HDO).

Another normalization approach is to perform a baseline scan before glucose ingestion and/or repetitive scans after glucose ingestion [54]. This could have enabled true probing of metabolic flux and thus improved atlas robustness by revealing intersubject differences in the timing of regional glucose metabolism. Yet, in the interest of time and simplicity, we chose not to add any extra scans, as this would have complicated the experimental setup and weakened the prospects of extending the use of DMI into larger studies and ultimately into clinical use. Instead, we aimed to perform a single DMI scan at the time point after glucose ingestion that corresponds to maximal glucose levels as discovered in previous studies [55].

In our age analysis, we found a 13% ± 4% decrease per decade in global Glx production which is close to the 9% ± 4% decrease per decade in bicarbonate production reported by Uthayakumar et al. [56]. When Uthayakumar et al. discovered that bicarbonate production decreases as much with age as lactate production does, they were surprised, because it contradicts the hypothesis of Goyal et al. stating that the known increase in oxygen–glucose index with age is mainly due to decreased lactate formation in aerobic glycolysis [47]. With our findings backing those of Uthayakumar et al., it supports the theory that the increased oxygen–glucose index is not merely caused by a decreased activity in non‐oxidative glucose pathways (e.g., aerobic glycolysis), but also by an increase in the oxidative metabolism of alternative energy substrates such as ketones.

Regarding lactate, our results differed from those of Uthayakumar et al. because we found no consistent distribution pattern and no age‐related changes in lactate production, except for an isolated increase in the occipital lobes. As discussed in the section on limitations, interpretations of lactate measures in DMI should be made with caution due to uncertainties in quantification. Furthermore, even if DMI lactate quantifications were reliable, our study might not be able to mirror the age‐related changes found by Uthayakumar et al. because of the combination of two factors: our cohort was older, and we used linear models. According to Goyal et al., the regional differences in aerobic glycolysis fade with age, and the age dependency of aerobic glycolysis is biphasic in some brain regions where an initial decrease is followed by an increase later in life [47]. Linear models cannot naturally capture a biphasic pattern, but due to the limited sample size in the current study, the use of nonlinear models (e.g., polynomials or generalized additive models with spline terms) would inevitably have led to overfitting, so studies with a much larger sample size are required for such analyses.

Since our atlas is to serve as a normative reference, we exemplified its use for patients with AD. This comparison was included as a proof‐of‐concept example of atlas application rather than an assessment of diagnostic accuracy. Compared with the normative atlas, patients with AD presented with less variation in the relative distribution of Glx measured as z‐scores. Z‐scores are a useful tool for assessing alterations in the region‐specific distribution of glucose metabolites, but cannot reveal any global alterations that affect all brain regions equally. The model for Glx production, on the other hand, revealed a global reduction in AD brains including all regions except the cerebellum. This resembles the findings of Khan et al. who used DMI to unveil a significantly reduced global Glx to glucose ratio in AD compared with HC [20]. Our specific uncovering of the cerebellum as normal is no surprise because this region is often spared in AD and for that very reason, used for normalization in FDG PET readings [57]. This compliance with current literature increases the reliability of the model.

However, it should be emphasized that the observed differences were primarily evident at the group level, whereas individual‐level distinctions were less pronounced. For instance, in the test dataset, a 76‐year‐old male HC exhibited a Glx profile resembling that of the AD group, while a 70‐year‐old female with AD showed values largely within the expected range for healthy subjects (Figure 4a). These individual deviations may partly reflect model limitations or biological variability. While alternative explanations such as undiagnosed preclinical AD in the HC or a very mild disease stage in the AD subject are possible, such interpretations cannot be directly assessed from the present data. Overall, these findings underscore that DMI should not be regarded as a standalone diagnostic tool. Similar to FDG PET, which is not used as the sole basis for AD diagnosis, DMI should be considered one component within a comprehensive clinical and paraclinical framework [11].

Proper discrimination between health and disease groups was only rendered possible after additional adjustments for weight, blood glucose increase, and waiting time from glucose ingestion to DMI scan. This suggests that although brain region, age, and sex explain some of the intersubject variability in Glx and lactate production, other variables might be stronger predictors. Since several of the variables are correlated (e.g., age and waiting time; sex and weight) (Figure S3), it could be a matter of confounding. While 30 healthy participants proved sufficient for detecting age‐related decline in Glx production with adequate statistical power (86%), the limited sample size restricts the number of variables to be reliably included in a model without overfitting. When estimating linear regression models, Green suggests a minimum of about 15 subjects per variable [58], and a potential prediction model for clinical use should be based on logistic regression, where 10–20 subjects per predictor are often needed [59]. In our study, this corresponds to a variable limit of 2–3, which was reached in the parsimonious models, leaving no room for additional adjustments. It is worth noticing that the additional adjustments included in the comprehensive models did not severely affect model complexity as judged by the minimal differences in AIC between the parsimonious and comprehensive models (Table S2). Still, the exact interplay and effects of the variables must be investigated in larger studies which could similarly explore the possible age–region interaction in lactate production discovered during model building. Thus, even if the present atlas captures major sources of biological variability, extension to broader populations and clinical use will require larger cohorts to support more complex modeling.

For such future studies, we recommend a protocol setup with standardization of as many variables as is feasible and practical in a clinical setting (at least 4 h of fasting, fixed dose of 75 g of glucose, minimal activity during waiting time), whereas non‐modifiable variables (participant weight, blood glucose increase) and variables difficult to control precisely in a clinical setting handling potentially demented patients (time from glucose ingestion to DMI scan) should instead be measured as part of the protocol setup to be systematically incorporated into the normative models. To further reduce the impact of random intersubject variability such as gastric emptying, future studies might favor intravenous over oral glucose administration [60]. However, the benefits of this choice must be weighed against the practical downsides.

Besides the limited sample size, other major limitations of this study are the technical challenges of DMI related to its inherently low signal‐to‐noise ratio (SNR), which constrains the spatial resolution. Improved SNR and spatial resolution can be achieved by increasing the magnetic field strength, but since all MRI scanners in routine clinical use are 3 T or below [61], we retained clinical relevance by not going above this.

Comparison with DMI studies using ultra‐high field strengths for improved spatial resolution leads to somewhat contradictory results for deuterated water, as some studies show low levels in the central brain regions [62, 63] while another shows high levels [64]. Results for Glx are more consistent because several ultra‐high field DMI studies report higher Glx in the occipital lobes than the frontal lobes [63, 64, 65], thereby mirroring the findings of our study. With respect to lactate, it is the glucose metabolite with the lowest SNR, and on top of that, its signal overlaps with that of lipids (Figure S2). Several ultra‐high field DMI studies find the highest lactate/lipid signals within the skull and subcutis or in the brain regions closest to these tissues [54, 62, 63, 64] which agrees with our results showing the highest lactate values (both as z‐scores and ratios) in the brain regions with close relation to the skull (Figures 2a and 3a). This supports the hypothesis of Ruhm et al. that lipid is the primary contributor to the signal [54]. Thus, the lactate signal is linked with large uncertainties in spectral fitting and probable lipid contamination, providing an additional reason for the general lack of significant associations for lactate in our study.

Finally, as we did not test the cognitive or physical condition of our healthy subjects, we cannot rule out undiagnosed diseases. Notably, the relatively high blood glucose of about 11–12 mmol/L measured in some of the healthy subjects ˜50 min after glucose ingestion is reason to believe that if blood glucose had been measured after 2 h as done in an oral glucose tolerance test, it may have been above the threshold for diabetes (11.1 mmol/L) or prediabetes (7.8 mmol/L) [66]. However, such results are likely to have been false positives since the reliability of the oral glucose tolerance test is currently under debate due to its dependence on random intersubject variabilities such as gastric emptying [60].

## 5 Conclusions

This study provides a vital step for DMI in the clinical translation. We established a normative reference atlas of brain glucose metabolism and found it to be greatly influenced by age and brain region but less by sex. With increasing age, a decrease in the glutamate and glutamine production was observed, suggesting a gradual reduction of oxidative phosphorylation. Measures of lactate production indicated higher aerobic glycolysis rates in males than females, but this was not significant after regional adjustment. Evidence was found that proper intersubject comparisons require additional adjustment for weight, blood glucose increase, and time from glucose ingestion to DMI scan. While the effect of these additional adjustments remains to be confirmed in larger studies, they enabled discrimination between healthy subjects and patients with AD who showed impaired oxidative phosphorylation. Together, these findings demonstrate that DMI‐based normative atlases can support physiologically informative intersubject comparisons, provided that key biological and acquisition‐related covariates are accounted for.

## Funding

This work was supported by Novo Nordisk Fonden (0084906), Lundbeck Foundation (R272‐2017‐4023).

## Conflicts of Interest

Rolf F. Schulte and Michael Vaeggemose are employees of GE HealthCare.

## Supporting information


**Table S1:** Minimum reporting standards in MR spectroscopy. CRLB: Cramér‐Rao lower bounds, MRSI: magnetic resonance spectroscopic imaging, FOV: field of view, DHO: deuterated water, Glx: glutamate plus glutamine, Glc: glucose, Lac: lactate, SNR: signal‐to‐noise ratio, AMARES: Advanced Method for Accurate, Robust, and Efficient Spectral fitting, MNS: Multi‐Nuclei Spectroscopy, MICO: Multiplicative intrinsic component optimization, tMPPCA: tensor Marchenko‐Pastur principal component analysis. * nominal matrix size = as to use the maximum k‐space extend not discarding information, effective matrix size = as to yield the same effective resolution as Cartesian encoding according to Rayleigh criterion at 64% height in the spatial domain.
**Table S2:** Model building. Building the models for glutamate plus glutamine (Glx) and lactate normalized to glucose and water was an iterative process, exploring the effect of segment, age, and sex. Although not part of the model building, the comprehensive models (highlighted in blue) are listed for comparison. In the ANOVA comparisons, the models were compared with the previously best model (highlighted in green), meaning that model 1 was compared with a model with no predictors, models 2–3 compared with model 1, and models 4–10 compared with model 3. (a) For the Glx model, including brain region as a predictor was significantly better than no predictors. Adding sex did not improve the model, but adding age did. No interaction terms improved the model. Thus, the final parsimonious model included only brain region and age with no interaction terms. (b) Similarly, for the lactate model, brain region significantly improved the model. Sex contributed substantially to explained variance, and although the sex term was only near significant, it was judged better than a model with only brain region. A region‐age interaction term significantly changed the model in ANOVA comparison and contributed substantially to explained variance, but also substantially worsened the AIC suggesting a high cost regarding model complexity. Therefore, the model with only brain region and sex and no interaction terms was chosen as the final parsimonious model for lactate. AIC: Akaike information criterion, Marginal *R* ^2^: variance explained only by the fixed effects not including the random effects.
**Figure S1:** Processed vs. raw spectra. Representative voxel spectra throughout the brain of a healthy volunteer. Each plotted voxel shows the magnitude signal of both the raw and processed data. Raw spectra are without denoising, partial volume correction and MICO bias field correction. Processed spectra colors correspond to the voxels of the colored rings in the DMI slice. The signal in the upper corners of the DMI slice originates from deuterated water phantoms.
**Figure S2:** Representative fits of parcellated spectra into regions. Examples from a single healthy volunteer. Each plot shows the real part of the phased region spectrum as well as the five fitted metabolites: DHO, glucose, glx, lactate, and lipid. The green line corresponds to the residual, i.e., the discrepancy between region spectrum and the sum of the five metabolites. R, right; L, left.
**Figure S3:** Correlation matrix. Pearson correlation coefficients for variables with a biologically plausible relation to glutamate plus glutamine (Glx) and lactate normalized to glucose (Glc) and water (HDO). Sex is set as a numeric factor with male as the reference. In the exploratory analysis, this correlation matrix was generated to identify additional variables with a correlation of at least 0.25 to be included in a comprehensive model: weight, blood glucose increase, and waiting time from glucose ingestion to DMI scan (Time to DMI).
**Figure S4:** Internal performance. For the 15 healthy females (blue) and the 15 healthy males (red) included in the normative atlas, the predicted values are plotted against the observed. The predicted values from both the parsimonious and the comprehensive models are shown for (a) glutamate plus glutamine (Glx) and (b) lactate. The black dotted lines are the identity lines. While the comprehensive model for Glx performs substantially better than the parsimonious model, the models for lactate perform almost equally. MAE: mean absolute error, L, left; R, right.

## Data Availability

Models and example code are openly available in the public repository “DMI normative brain ATLAS” at https://doi.org/10.6084/m9.figshare.31241707. Raw data on DMI is not publicly available to preserve individuals' privacy under the European General Data Protection Regulation.
