# Supplementary material for: Atlas of Brain Glucose Metabolism Using Deuterium Metabolic Imaging at 3 T
Source: Magn Reson Med. 2026 May 30;96(4):1834–45. doi: 10.1002/mrm.70451 (PMC13419004; doi:10.1002/mrm.70451)
Supplement: Supplementary file 1 — Table S1: Minimum reporting standards in MR spectroscopy. CRLB: Cramér‐Rao lower bounds, MRSI: magnetic resonance spectroscopic imaging, FOV: field of view, DHO: deuterated water, Glx: glutamate plus glutamine, Glc: glucose, Lac: lactate, SNR: signal‐to‐noise ratio, AMARES: Advanced Method for Accurate, Robust, and Efficient Spectral fitting, MNS: Multi‐Nuclei Spectroscopy, MICO: Multiplicative intrinsic component optimization, tMPPCA: tensor Marchenko‐Pastur principal component analysis. * nominal matrix size = as to use the maximum k‐space extend not discarding information, effective matrix size = as to yield the same effective resolution as Cartesian encoding according to Rayleigh criterion at 64% height in the spatial domain. Table S2: Model building. Building the models for glutamate plus glutamine (Glx) and lactate normalized to glucose and water was an iterative process, exploring the effect of segment, age, and sex. Although not part of the model building, the comprehensive models (highlighted in blue) are listed for comparison. In the ANOVA comparisons, the models were compared with the previously best model (highlighted in green), meaning that model 1 was compared with a model with no predictors, models 2–3 compared with model 1, and models 4–10 compared with model 3. (a) For the Glx model, including brain region as a predictor was significantly better than no predictors. Adding sex did not improve the model, but adding age did. No interaction terms improved the model. Thus, the final parsimonious model included only brain region and age with no interaction terms. (b) Similarly, for the lactate model, brain region significantly improved the model. Sex contributed substantially to explained variance, and although the sex term was only near significant, it was judged better than a model with only brain region. A region‐age interaction term significantly changed the model in ANOVA comparison and contributed substantially to explained variance, but also substa [file MRM-96-1834-s001.docx]

# Supplementary Material for Review

| **Table S1 Minimum Reporting Standards in MR Spectroscopy**  *in accordance with Lin et al. NMR Biomed 2021* | |
| --- | --- |
| **1. Hardware** |  |
| *a. Field strength [T]* | 3T |
| *b. Manufacturer* | GE HealthCare |
| *c. Model (software version if available)* | DV_26_R03 |
| *d. RF coils: nuclei (transmit/ receive), number of channels, type, body part* | ^2^H/^1^H dual tuned quadrature birdcage head coil, transmit/receive, 1 channel, (PulseTeq, Surrey, UK) |
| *e. Additional hardware* | ^1^H 32 channel head coil (GE HealthCare, Waukesha, WI, USA) |
| **2. Acquisition** |  |
| *a. Pulse sequence* | 3D density-weighted MRSI |
| *b. Volume of Interest (VOI) locations* | whole brain, soft pulse (pulse width is 1800 us) with 70° flip angle. |
| *c. Nominal VOI size [cm^3^, mm^3^]* | FOV 240x240x240 mm^3^ |
| *d. Repetition Time (TR), Echo Time (TE) [ms, s]* | TR = 155.8 ms, TE = 0.7 ms |
| *e. Total number of Excitations or acquisitions per spectrum* | 1678, 4 repetitions (17:26 min acquisition time) |
| *In time series for kinetic studies* |  |
| *i.         Number of Averaged spectra (NA) per time-point* | 4 |
| *ii.       Averaging method (e.g. block-wise or moving average)* | - |
| *iii.      Total number of spectra (acquired / in time-series)* | - |
| *f. Additional sequence parameters (spectral width in Hz, number of spectral points, frequency offsets); If STEAM: Mixing Time TM; If MRSI: 2D or 3D, FOV in all directions, matrix size, acceleration factors* | BW = 5 kHz  Number of spectral points = 700  Matrix size = 16x16x16 nominal, 10x10x10 effective* |
| *g. Water Suppression Method* | - |
| *h. Shimming Method, reference peak, and thresholds for “acceptance of shim” chosen* | Second-order shimming was employed, and a B0 map (IDEAL IQ, 2 × 2 × 2 mm3, repetition time 6.9 ms, echo train length 3, echo time 1–5.2 ms). Adjustment, ^1^H RMS current values < 20 mA, Region: whole brain |
| *i. Triggering or motion correction method (respiratory, peripheral, cardiac triggering, incl. device used and delays)* | - |
| **3. Data analysis methods and outputs** |  |
| *a. Analysis software* | MNS Research Pack (GE HealthCare)  Denoised using tMPPCA (DOI: 10.1002/mrm.29817)  Bias field correction using the MICO (doi: 10.1016/j.mri.2014.03.010)  MRSI metabolite fitting using OXSA-AMARES (https://doi.org/10.1371/journal.pone.0185356)  Parcellation of co-registered metabolic MRSI and anatomical T1 weighted images using SPM12 (ISBN-10: 0-12-372560-7) |
| *b. Processing steps deviating from quoted reference or product* | - |
| *c. Output measure (e.g. absolute concentration, institutional units, ratio)* | Ratio |
| *d. Quantification references and assumptions, fitting model assumptions* | For probing the conversion of glucose into products of oxidative phosphorylation (Glx) and aerobic glycolysis (lactate), the signals of each of these metabolic products were normalized to the combined glucose and HDO peaks. The combined signal was chosen (as opposed to e.g. only the glucose peak) to circumvent potential separation errors caused by the fact that glucose and HDO signals are spectrally overlapping, thus making the combined peaks a more robust normalization reference. |
| **4. Data Quality** |  |
| *a. Reported variables (SNR, Linewidth (with reference peaks))* | AMARES fitting was used to determine SNR, linewidth, chemical shift, and Cramér–Rao lower bounds (CRLBs) across all voxels with sufficient SNR (HDO SNR > 5) |
| *b. Data exclusion criteria* | Line width > 30 Hz and CRLBs > 50 % for DHO, Glc, Glx, Lac. |
| *c. Quality measures of postprocessing Model fitting (e.g. CRLB, goodness of fit, SD of residual)* | - |
| *d. Sample Spectrum* | - |

**Table S1 | Minimum Reporting Standards in MR Spectroscopy**. CRLB: Cramér-Rao lower bounds, MRSI: magnetic resonance spectroscopic imaging, FOV: field of view, DHO: deuterated water, Glx: glutamate plus glutamine, Glc: glucose, Lac: lactate, SNR: signal-to-noise ratio, AMARES: Advanced Method for Accurate, Robust, and Efficient Spectral fitting, MNS: Multi-Nuclei Spectroscopy, MICO: Multiplicative intrinsic component optimization, tMPPCA: tensor Marchenko-Pastur principal component analysis. * nominal matrix size = as to use the maximum k-space extend not discarding information, effective matrix size = as to yield the same effective resolution as Cartesian encoding according to Rayleigh criterion at 64% height in the spatial domain.

| **Table S2 Model building** | | | | |
| --- | --- | --- | --- | --- |
| 1. **Glx** | | | | |
| No. | Model | ANOVA p-value | AIC | Marginal R^2^ |
| **1** | **Region** | **<10^-15^** | **-1433** | **0.094** |
| 2 | Region + Sex | 0.662 | -1425 | 0.096 |
| **3** | **Region + Age** | **0.029** | -1423 | **0.184** |
| 4 | Region + Age + Sex | 0.720 | -1414 | 0.183 |
| 5 | Region * Sex | 0.637 | -1334 | 0.104 |
| 6 | Region * Age | 0.248 | -1264 | 0.192 |
| 7 | Region * Age + Sex | 0.310 | -1255 | 0.191 |
| 8 | Region * Sex + Age | 0.332 | -1323 | 0.190 |
| 9 | Region * Age + Age * Sex | 0.384 | -1242 | 0.187 |
| 10 | Comprehensive model:  Region + Age + Weight + Blood glc increase + Time to DMI | **0.046** | -1390 | **0.297** |
| 1. **Lactate** | | | | |
| No. | Model | ANOVA p-value | AIC | Marginal R^2^ |
| **1** | **Region** | **<10^-15^** | **-1392** | **0.175** |
| 2 | Region + Age | 0.333 | -1378 | 0.185 |
| **3** | **Region + Sex** | 0.063 | -1387 | **0.219** |
| 4 | Region + Age + Sex | 0.353 | -1372 | 0.226 |
| 5 | Region * Sex | 0.268 | -1298 | 0.230 |
| 6 | Region * Age | **0.023** | -1232 | 0.209 |
| 7 | Region * Age + Sex | **0.012** | -1226 | **0.249** |
| 8 | Region * Sex + Age | 0.284 | -1283 | 0.238 |
| 9 | Region * Sex + Sex * Age | 0.355 | -1269 | 0.234 |
| 10 | Comprehensive model:  Region + Sex + Weight + Blood glc increase + Time to DMI | 0.608 | -1347 | 0.231 |

**Table S2 | Model building**. Building the models for glutamate plus glutamine (Glx) and lactate normalized to glucose and water was an iterative process, exploring the effect of segment, age, and sex. Although not part of the model building, the comprehensive models (highlighted in blue) are listed for comparison. In the ANOVA comparisons, the models were compared with the previously best model (highlighted in green), meaning that model 1 was compared with a model with no predictors, models 2-3 compared with model 1, and models 4-10 compared with model 3. **a)** For the Glx model, including brain region as a predictor was significantly better than no predictors. Adding sex did not improve the model, but adding age did. No interaction terms improved the model. Thus, the final parsimonious model included only brain region and age with no interaction terms. **b)** Similarly, for the lactate model, brain region significantly improved the model. Sex contributed substantially to explained variance, and although the sex term was only near significant, it was judged better than a model with only brain region. A region-age interaction term significantly changed the model in ANOVA comparison and contributed substantially to explained variance, but also substantially worsened the AIC suggesting a high cost regarding model complexity. Therefore, the model with only brain region and sex and no interaction terms was chosen as the final parsimonious model for lactate. AIC: Akaike information criterion, Marginal R^2^: variance explained only by the fixed effects not including the random effects

**
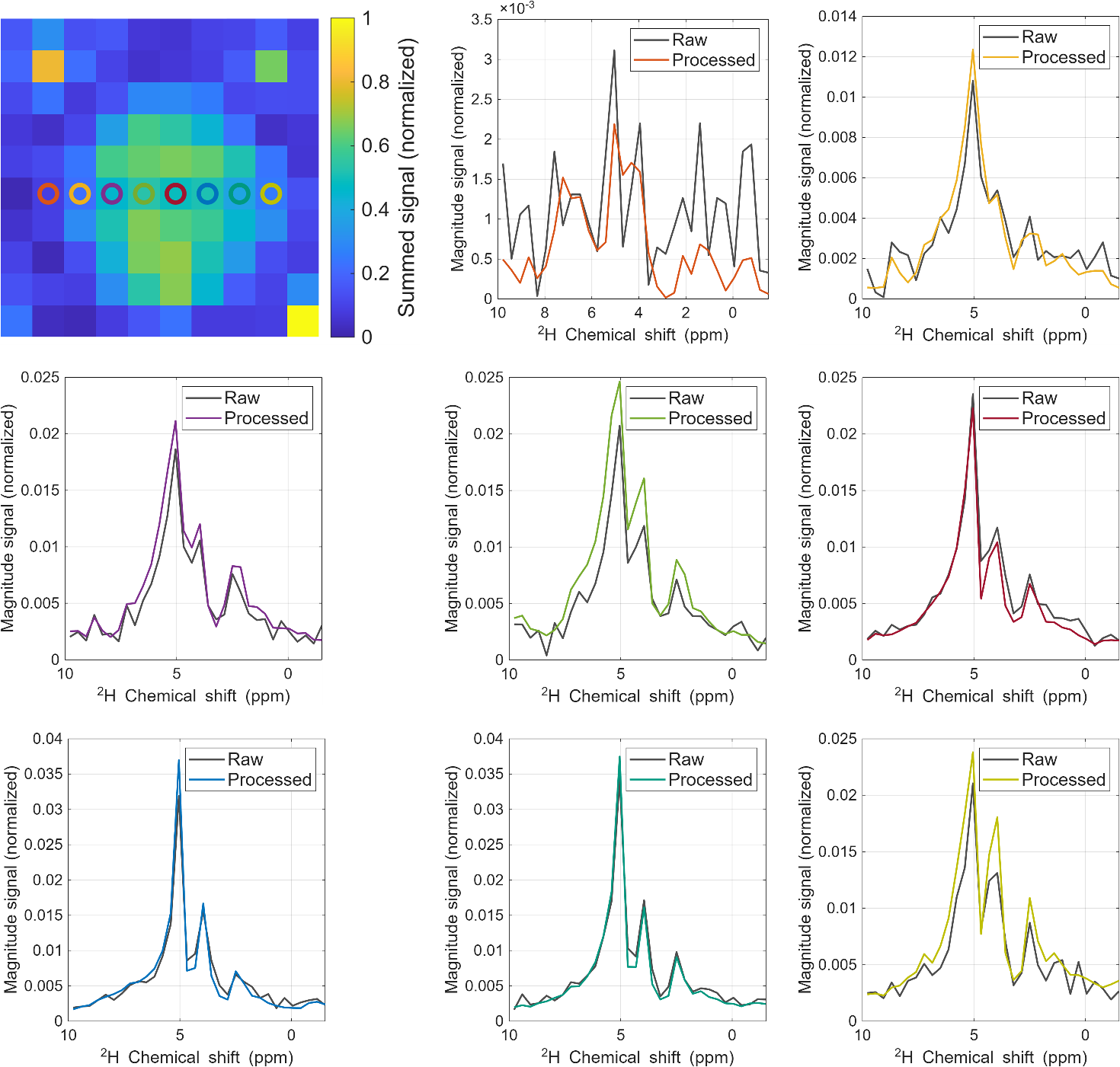
**

**Figure S1 | Processed vs. raw spectra.** Representative voxel spectra throughout the brain of a healthy volunteer. Each plotted voxel shows the magnitude signal of both the raw and processed data. Raw spectra are without denoising, partial volume correction and MICO bias field correction. Processed spectra colours correspond to the voxels of the coloured rings in the DMI slice. The signal in the upper corners of the DMI slice originates from deuterated water phantoms.

**
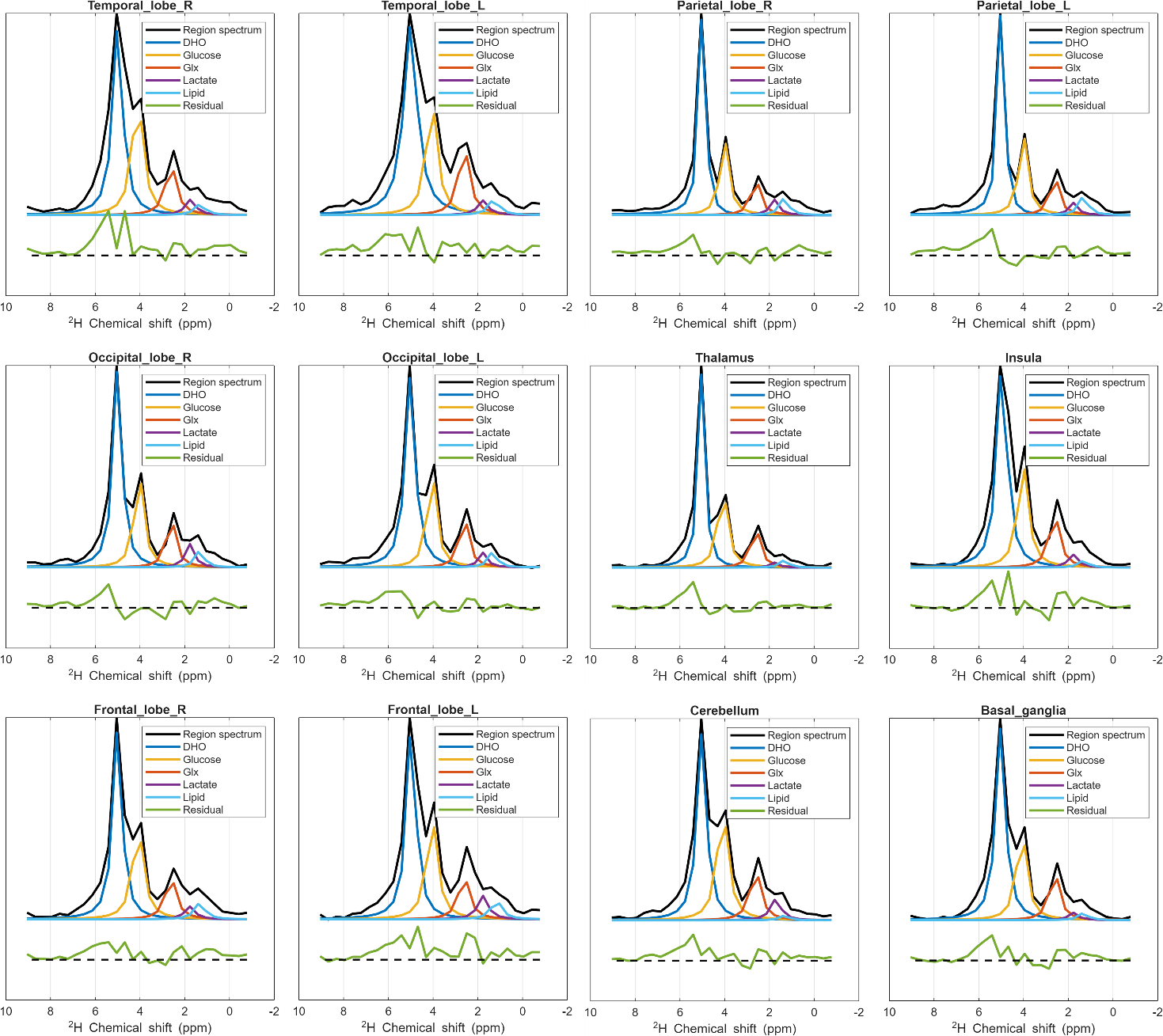
**

**Figure S2 | Representative fits of parcellated spectra into regions.** Examples from a single healthy volunteer. Each plot shows the real part of the phased region spectrum as well as the five fitted metabolites: DHO, glucose, glx, lactate and lipid. The green line corresponds to the residual, i.e. the discrepancy between region spectrum and the sum of the five metabolites. R: right, L: left.


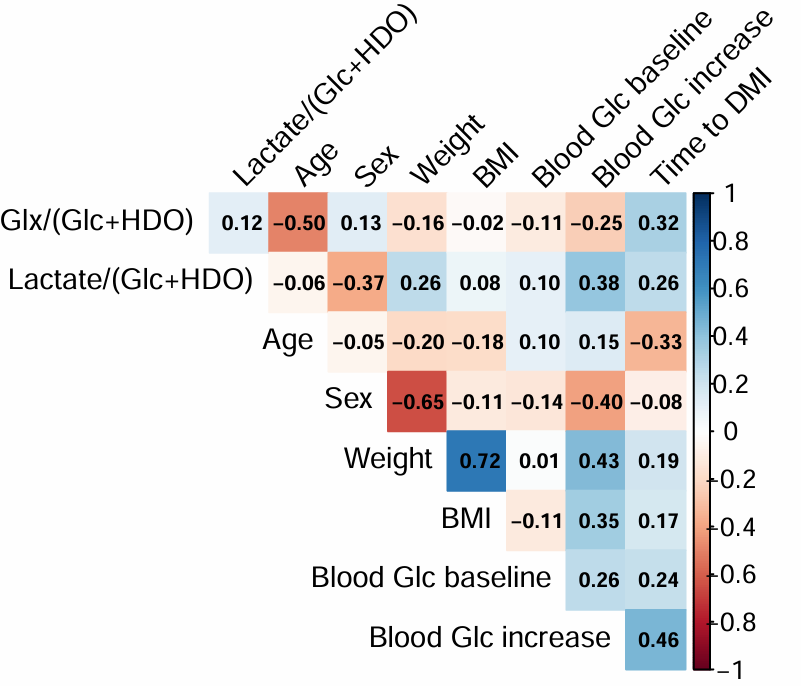


**Figure S3 | Correlation matrix**. Shows Pearson correlation coefficients for variables with a biologically plausible relation to glutamate plus glutamine (Glx) and lactate normalized to glucose (Glc) and water (HDO). Sex is set as a numeric factor with male as the reference. In the exploratory analysis, this correlation matrix was generated to identify additional variables with a correlation of at least 0.25 to be included in a comprehensive model: weight, blood glucose increase, and waiting time from glucose ingestion to DMI scan (Time to DMI).

**
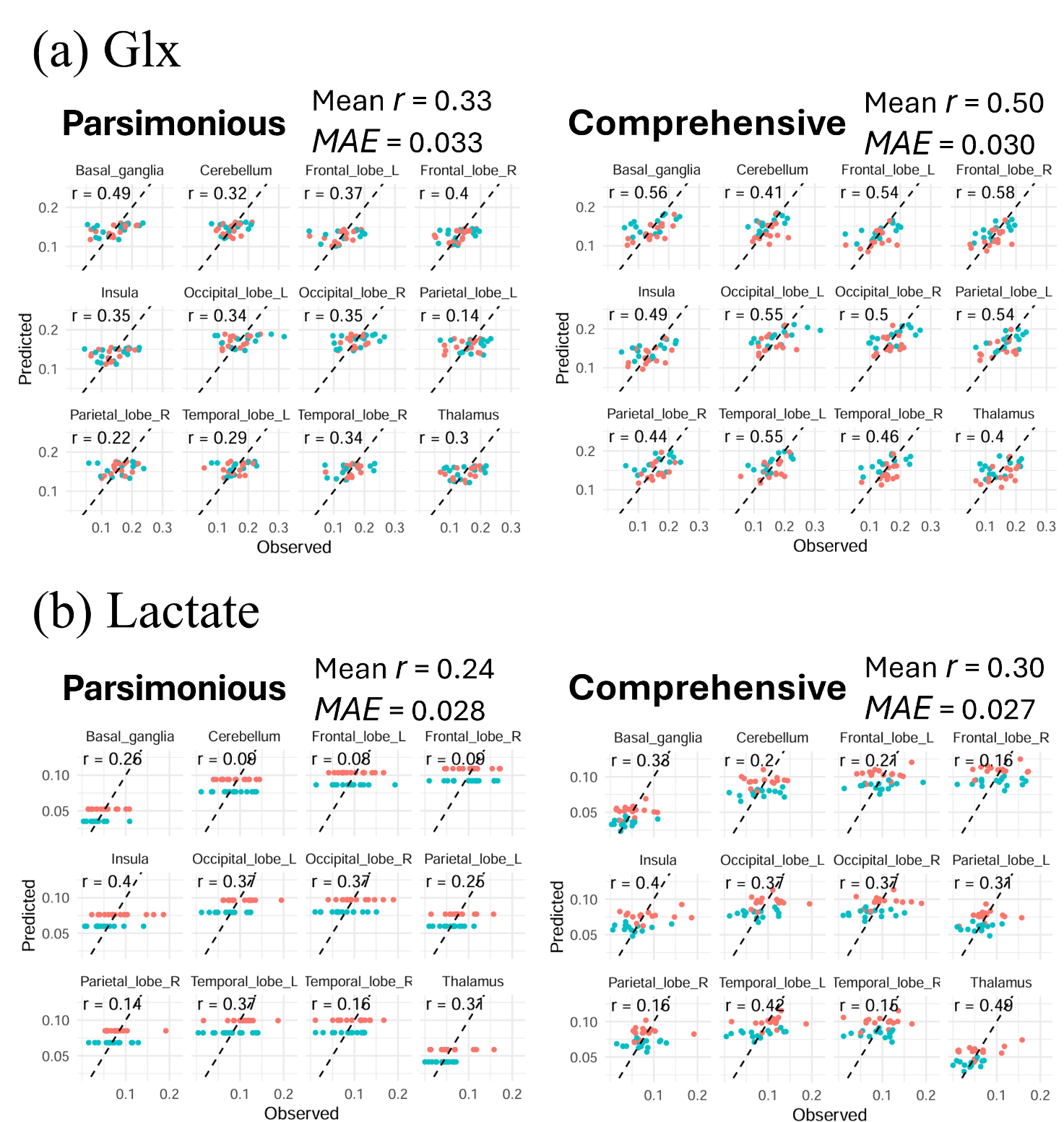
**

**Figure S4 | Internal performance.** For the 15 healthy females (blue) and the 15 healthy males (red) included in the normative atlas, the predicted values are plotted against the observed. The predicted values from both the parsimonious and the comprehensive models are shown for **a)** glutamate plus glutamine (Glx) and **b)** lactate. The black dotted lines are the identity lines. While the comprehensive model for Glx performs substantially better than the parsimonious model, the models for lactate perform almost equally. MAE: mean absolute error, L: left, R: right
